# Type II Natural Killer T Cells Contribute to Protection Against Systemic Methicillin-Resistant *Staphylococcus aureus* Infection

**DOI:** 10.3389/fimmu.2020.610010

**Published:** 2020-11-18

**Authors:** Samantha Genardi, Lavanya Visvabharathy, Liang Cao, Eva Morgun, Yongyong Cui, Chao Qi, Yi-Hua Chen, Laurent Gapin, Evgeny Berdyshev, Chyung-Ru Wang

**Affiliations:** ^1^ Department of Microbiology and Immunology, Northwestern University Feinberg School of Medicine, Chicago, IL, United States; ^2^ Department of Pathology, Northwestern University Feinberg School of Medicine, Chicago, IL, United States; ^3^ Department of Immunology and Microbiology, University of Colorado School of Medicine, Aurora, CO, United States; ^4^ Department of Medicine, National Jewish Health, Denver, CO, United States

**Keywords:** *Staphylococcus aureus*, natural killer T cells, CD1, lipid antigens, cytokine, knockout mice

## Abstract

Methicillin-resistant *Staphylococcus aureus* (SA) bacteremia is responsible for over 10,000 deaths in the hospital setting each year. Both conventional CD4^+^ T cells and γδ T cells play protective roles in SA infection through secretion of IFN-γ and IL-17. However, the role of other unconventional T cells in SA infection is largely unknown. Natural killer T (NKT) cells, a subset of innate-like T cells, are activated rapidly in response to a wide range of self and microbial lipid antigens presented by MHC I-like molecule CD1d. NKT cells are divided into two groups, invariant NKT (iNKT) and type II NKT cells, based on TCR usage. Using mice lacking either iNKT cells or both types of NKT cells, we show that both NKT cell subsets are activated after systemic SA infection and produce IFN-γ in response to SA antigen, however type II NKT cells are sufficient to control bacterial burden and inflammatory infiltrate in infected organs. This protective capacity was specific for NKT cells, as mice lacking mucosal associated invariant T (MAIT) cells, another innate-like T cell subset, had no increased susceptibility to SA systemic infection. We identify polar lipid species from SA that induce IFN-γ production from type II NKT cells, which requires both CD1d-TCR engagement and IL-12 production by antigen presenting cells. We also demonstrate that a population of T cells enriched for type II NKT cells are increased in PBMC of SA bacteremic patients compared to healthy controls. Therefore, type II NKT cells perform effector functions that enhance control of SA infection prior to conventional T cell activation and recognize SA-derived lipid antigens. As CD1d is highly conserved in humans, these CD1d-restricted SA lipid antigens could be used in the design of next generation SA vaccines targeting cell-mediated immunity.

## Introduction

*Staphylococcus aureus* (SA) is a leading cause of healthcare-associated and community-acquired infection in the United States. SA causes a range of infections in humans, including skin and soft tissue infection (SSTI), pneumonia, endocarditis, and bacteremia, which if left untreated, can lead to sepsis and high levels of mortality (1). Despite the traditional thinking that humoral immunity is the main driver of immune defense against extracellular pathogens, T cells are now recognized as critical players in protection against SA in multiple routes of infection, as shown in humans and preclinical animal models. HIV patients with decreased CD4^+^ T cell counts have increased susceptibility to SA bacteremia (2, 3). Additionally, patients with hyper IgE syndrome who have a STAT3 mutation resulting in an inability to develop Th17 cells have increased susceptibility to SA skin and pulmonary infections (4). In mouse models of infection, both CD4^+^ T cells and γδ T cells produce cytokines and protect against SA. CD4^+^ memory T cells produce IFN-γ upon secondary peritonitis challenge and promote recruitment of macrophages and clearance of SA (5). In the skin, γδ T cell production of IL-17A is necessary for neutrophil recruitment to sites of infection and decreased bacterial burden (6). A recent study demonstrates that clonotypic Vγ6^+^Vδ4^+^ T cells are the primary source of IL-17-producing T cells which drive protection in mouse SA skin infection (7).

While conventional CD4^+^ T cells and γδ T cells have been studied in SA infection, the role of other non-conventional T cell subsets in SA infection is less explored. Natural killer T (NKT) cells are innate resident T lymphocytes that are activated early in response to infection, and rapidly secrete a wide range of cytokines, depending on the nature of the stimuli (8). NKT cells are restricted by the MHC class I-like molecule CD1d, which presents lipid antigens rather than peptide antigens, and can be divided into two groups based on TCR usage and lipid antigen recognition (9, 10). Invariant (iNKT) cells recognize the lipid agonist α-galactosylceramide (α-GalCer), which, when loaded onto a CD1d tetramer, can be used to identify this population *in vivo* (11–13). iNKT cells also recognize glycosphingolipids from *Sphingomonas* species (14) and glycoglycerol lipids from *Streptococcus pneumoniae (S. pneumoniae)* and *Borrelia burgdorferi* (15). In mouse models of *S. pneumoniae* and *Borrelia burgdorferi* infection, rapid cytokine production by iNKT cells recruited innate immune cells to the site of infection and contributed to bacterial clearance (16, 17). While iNKT cells are the dominant NKT cell subset in mice, they make up a minority of the NKT cell pool in humans, with type II NKT cells being the dominant NKT cell subset (18). Type II NKT cells express a more diverse TCR repertoire and recognize a wide range of self and microbial lipid antigens. Due to the lack of specific tools to identify this polyclonal population *in vivo*, type II NKT cells have been understudied compared to iNKT cells, though some studies have demonstrated an active role for type II NKT cells during infection (19). In a mouse model of SA sepsis, Cardell and colleagues showed that administration of sulfatide, a self-lipid known to stimulate a subset of type II NKT cells, protected mice from lethal SA systemic challenge (20). Type II NKT cells also recognize phosphatidylglycerol (PG), cardiolipin, and phosphatidylinositol from *Corynebacterium* *glutamicum* (*C. glutamicum*) and *Mycobacterium tuberculosis (Mtb)*, and PG from *Listeria monocytogenes (L. monocytogenes)* (21, 22). Both subsets of NKT cells can have synergistic or opposing actions in models of infection. In *Trypanosoma cruzi* infection, type II NKT cells drove a proinflammatory phenotype that increased parasite-induced mortality and decreased generation of pathogen-specific antibodies, whereas iNKT cells were anti-inflammatory and contributed to lowered mortality (23). In contrast, both iNKT and type II NKT cells protected mice from hepatitis B virus (HBV) infection, but their mechanisms of action were different; type II NKT cells were activated by CD1d-presented HBV modified lysophospholipids while iNKT cells were activated indirectly by secreted IL-12 (24). These studies highlight the differential role of NKT cell subsets in response to various pathogens. However, the relative contribution of NKT cell subsets to protective immunity against SA infection has not been explored.

In this study, we determined whether iNKT or type II NKT cells play dominant or synergistic roles during systemic methicillin-resistant SA infection and whether these T cells subsets can recognize antigens derived from SA that could be used in the design of a subunit vaccine. Our data shows that both NKT cell subsets are activated and expanded after SA infection. However, only type II NKT cells are necessary for lowering bacterial burden in SA infected liver and kidneys at early times post-infection, mediated by IFN-γ production to polar lipid species derived from SA. This protective effect was unique for type II NKT cells, as mice lacking MAIT cells, another innate-like T cell subset that recognizes microbially derived vitamin B-related metabolites (25, 26), had no significant increase in SA bacterial burden. We also demonstrate that NKT-like cells, but not MAIT cells, are elevated in the PBMC of patients with systemic SA infection, demonstrating the relevance of our findings to the human setting.

## Materials and Methods

### Ethics Statement

This study was carried out in strict accordance with the recommendations in the Guide for the Care and Use of Laboratory Animals of the National Institutes of Health. The protocol was approved by the Animal Care and Use Committee of the Northwestern University (Protocol number: IS00001659). For human blood collection, the protocol was approved by the Northwestern Institutional Review Board (IRB #STU0001210512).

### Mice

Wildtype C57BL/6 (B6) mice and MHC class II deficient (MHC-II^-/-^) mice were obtained from The Jackson Laboratory (Bar Harbor, ME). MyD88^-/-^ mice were obtained from Mutant Mouse Resource and Research Centers. Jα18(-10)^-/-^ mice (hereafter referred to as Jα18^-/-^ mice) were generated on the B6 background in Dr. Laurent Gapin’s lab (University of Colorado) (27). We backcrossed these Jα18^-/-^ mice to B6 mice in our mouse colony to generate wild-type littermate controls. CD1d^-/-^ mice were generated in house and have been backcrossed onto B6 background for at least 12 generations (28). MR1^-/-^ mice were provided by Dr. Ted Hansen (Washington University) and backcrossed to B6 mice to generate MR1^+/+^ littermate controls. Vα3.2^+^Vβ9^+^ CD1d-autoreactive transgenic mice (24αβ) were provided by Dr. Suzanna Cardell (University of Gothenburg) (29). MHC-II^-/-^CD1d^-/-^ mice were generated by crossing CD1d^-/-^ mice and MHC-II^-/-^ mice. Naïve mice were housed in a specific pathogen-free facility.

### *S. aureus* Bacteremia Model and Bacterial Quantification

All data were obtained using the USA300 methicillin-resistant SA clinical isolate strain, generously provided by Dr. Nancy Freitag (University of Illinois at Chicago). Briefly, SA was grown up overnight in tryptic soy broth (MP Biomedicals, Solon, OH) at 37°C, 220 rpm, diluted 1:100 the next day, and grown to mid-log phase. Bacteria were wash, resuspended, and inoculated *via* tail vein with 2x10^6^ or 1x10^7^ CFU USA300 in PBS. For colony forming unit (CFU) quantification, whole liver, kidney, or spleen were homogenized in PBS by sonication, raised to a volume of 10 ml with PBS, and plated in serial dilutions on tryptic soy agar containing 5 µg/ml erythromycin.

### Histology Imaging

Hematoxylin and Eosin (H&E) staining of SA-infected kidneys were performed by the Northwestern mouse histology and phenotyping core. H&E slides were imaged using the TissueGnostics Imaging System and inflammatory foci were quantified using Tissue/HistoQuest software. Two sections from each kidney of each mouse were quantified and the average inflammatory foci for each mouse was calculated and recorded as one data point.

### *S. aureus* Lipid Isolation and Fractionation

SA lipid isolation and fractionation were performed as described previously (30). Briefly, bacterial pellet from the overnight culture of USA300 was treated with lysostaphin to lyse the peptidoglycan cell wall and release protoplasts. Total lipid was extracted from protoplasts using a modified Bligh-Dyer extraction method (31) and was fractionated using silica gel column chromatography and a chloroform-methanol gradient. Dominant lipid fractions were identified using thin layer chromatography, as previously described (30), and listed in **Figure 6A**.

### Mouse Cell Preparation and Lipid Pulsing

Mouse leukocyte single cell suspensions were isolated from lymph node (LN), liver, spleen, and kidney of naïve and infected mice. Lymphocytes were isolated from kidneys and livers using a 37.5% Percoll gradient centrifugation. For ELISPOT total fraction screening experiments (**Figure 5B**) single cell suspensions of Jα18^-/-^ liver were enriched for CD8^-^ T cells (as type II NKT cells are either CD4^+^ of DN) by magnetic-activated cell sorting (MACS) negative selection (Miltenyi Biotec), using biotinylated CD8α/β, B220, MHCII, ter119, Ly6G, and anti-biotin beads. For FR-8 ELISPOT and adoptive transfer experiments, we included additional mAb specific to TCRγδ and CD11b in the negative selection cocktail to further enriched for type II NKT cells. BMDCs were derived from mouse bone marrow progenitors in complete RPMI (cRPMI) supplemented with 10 ng/ml GM-CSF (PeproTech, 315–03) and 2 ng/ml IL-4 (PeproTech, 214–14). Mature BMDCs were pulsed with 10 µg/ml of total SA lipids (SAlip) or lipid fractions overnight. Total SA lipids and lipid fractions were prepared by drying down eluent buffer and resuspending at the appropriate concentration in cRPMI. All lipids were sonicated for 15 min in a water bath sonicator before pulsing.

### Human Sample Acquisition and Processing

PBMC were acquired through the Northwestern Memorial Hospital. The inclusion criteria for infected patients was as follows: patients with confirmed SA bacteremia (by at least one blood culture where blood has been collected by use of aseptic technique), patients who were inpatients at NMH at the time of study collection, patients over the age of 18, and patients able to give consent for blood collection. The exclusion criteria were as follows: pregnant individuals, mixed bacteremic patients, patients with known active infections with blood-borne viruses (including human immunodeficiency virus Ab/Ag positive, hepatitis C RNA positive, hepatitis B sAg positive, and SARS-CoV-2 RNA positive individuals), patients with active malignancies (including hematologic and solid organ malignancies), solid organ transplant recipients, patients with steroid treatment for at least 1 month (>30 mg/day), and patients on cytotoxic immunosuppressive therapy (including calcineurin inhibitors, e.g. cyclosporine, tacrolimus, antiproliferative agents, e.g. azathioprine, cyclophosphamide, methotrexate, chlorambucil, mycophenylate mofetil, and immune-active monoclonal antibodies, e.g. adalimumab, alemtuzumab, belimumab, golimumab, infliximab, muromonab-CD3, natalizumab, ofatumumab, rituximab, tocilizumab, tocitumomab). Patient demographics for consented individuals is as follows: N=9 healthy controls (21) (5 males, 4 females), N=7 SA bacteremic patients (SA) (four males, three females). The median age of HC was 31 ± 3.46 and the median age of SA was 60 ± 16.21. Of the SA patients, 1 had MRSA and 6 had MSSA. Each infected sample collected was paired with healthy donor blood for side by side processing and downstream assays. PBMC were isolated from whole blood within 48 h of original blood draw using Histopaque-1077 (Sigma-Aldrich). Leukocytes were collected at the serum, cell interface and washed 3x before counting cells with a hemocytometer and performing surface staining.

### Reagents and Antibodies

Mouse and human CD1d tetramer (TET^+^) unloaded or loaded with α-galactosylceramide (α-GalCer) analog PBS57 and human MR1 tetramer (TET^+^) loaded with 6-FP (control) or 5-OP-RU were provided by NIH tetramer facility. Fluorochrome-conjugated antibodies against mouse CD3, CD4, CD8α, CD69, NK1.1, TCRβ, Ly6G, CD11b, B220, CD11c, Ly6C, F4-80, IFN-γ, IL-17A, Vβ2, Vβ7, and Vβ12 and human CD3, CD4, CD8α, Vα7.2, CD161, CD14, and CD19 were purchased from BioLegend. Vβ4, Vβ5.1/5.2, Vβ6, Vβ8.1/8.2, Vβ9, Vβ11, and Vβ13 were purchased from BD. Anti-Vβ10 was purchased from eBioscience. Live dead eFlour506 was purchased from Thermo Fischer Scientific.

### Flow Cytometry

Single cell suspensions were incubated with purified 2.4G2 mAb or human Fc block (BD Biosciences) then stained with specific antibodies. For intracellular staining experiments, cells were stimulated with 50 ng/ml phorbol 12-myristate 13-acetate (32) (Enzo Life Sciences, BML-PE160-0005) and 0.5 µg/ml Ionomycin (Sigma, I9657-5MG) for 2 h followed by 4 h of brefeldin A (BioLegend, 420601) stimulation. Following stimulation, cells were surface stained, then fixed overnight with 0.5% paraformaldehyde (Sigma, 158127-3KG). The next day, cells were permeabilized with 0.05% saponin (Sigma, S7900), then intracellularly stained with anti-cytokine antibodies. Flow cytometry was performed on a FACSCanto II (BD Biosciences) and data analysis was performed with FlowJo Software version 10 (Tree Star, Inc.).

### Cytokine Secretion Assays

Enzyme-linked immunosorbant assay (ELISA) was performed to detect IFN-γ or IL-17A cytokine secretion. Cytometric bead assay (CBA) was performed to detect IL-4, IL-6, IL-10, IL-13, IL-23, TNF-α, and GM-CSF cytokine secretion. Single cell suspensions of liver or spleen were incubated with α-GalCer (200 ng/ml) or heat-killed SA (HKSA) (10^6^ CFU) for 48 h, then supernatant was collected for ELISA or CBA. For ELISPOT assay, Multiscreen-IP plates (Millipore) were coated with 10 µg/ml mouse anti-IFN-γ or anti-IL-17A overnight in PBS, then blocked with cRPMI. Total liver lymphocytes or T cell enriched liver lymphocytes were cultured with total SA lipid or SA lipid fraction-pulsed BMDCs for 18-20 h before assay development, detailed assay protocol published previously (33). Developed ELISPOT plates were imaged using an ImmunoSpot reader (Cellular Technology Ltd.).

### RNA Isolation and Real-Time PCR

T cells were enriched from Jα18^-/-^ mouse liver or spleen lymphocytes using MACS negative selection as previously described. Type II NKT cells were sorted from pooled enriched T cells using a BD FacsAria. RNA was extracted from sorted cells using RNA-easy kit (QIAGEN) and cDNA was generated using Superscript II reverse transcriptase (Invitrogen). RT-PCR was performed using SYBR Green PCR master mix with cytokine primers (listed in **Supplementary Table 1**) and MyiQ real-time detection system (Bio-Rad). PCR were run in duplicate and cytokine values were normalized to b-actin as housekeeping gene.

### Adoptive Transfer of Type II NKT Cells

Recipient mice (CD45.1^+^ mice) were irradiated with 900 rads of cesium 1 day prior to adoptive transfer. Splenocytes from CD45.2^+^24αβ Tg mice were enriched for T cells as previously described above. 5x10^6^ cells were transferred to recipient mice. 1 day after adoptive transfer, recipient mice were infected *via* tail vein with 2x10^6^ CFU of SA. Mice were euthanized at 2 dpi and organs were isolated for CFU quantification and intracellular staining.

### Statistical Analysis

All statistical analyses were performed using GraphPad Prism software. Unpaired Student’s *t* test was used for comparison of two groups, one-way ANOVA was used for comparison of more than two groups, and two-way ANOVA was used for comparison of more than two groups and two variables. For CFU quantification statistics, Mann-Whitney test was used. Values are represented as mean + SEM. Statistical significance is denoted by the annotation: *p<0.05; **p<0.01; ***p<0.001; ****p<0.0001.

## Results

### Mice Lacking NKT Cells but Not MAIT Cells Had Increased Bacterial Burden and Neutrophil Infiltration in Infected Liver and Kidneys Following Systemic SA Infection

**Figure 1:**
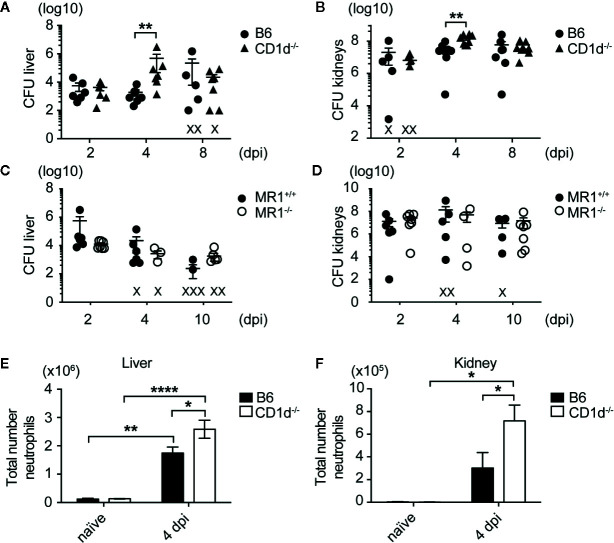
Mice lacking NKT cells have increased bacterial burdens and neutrophilic infiltrate in SA infected organs. **(A, B)** Colony forming units (CFU) quantified in liver **(A)** and kidneys **(B)** of B6 and CD1d^-/-^ mice at various times post infection (N=6–8 mice/genotype/timepoint). **(C, D)** CFU quantified in liver **(C)** and kidneys **(D)** of MR1^+/+^ and MR1^-/-^ mice at various times post infection (N=4-7 mice/genotype/timepoint). **(E, F)** Total number of neutrophils in the liver **(E)** and kidney **(F)** of infected mice (liver: N=3 naïve, N=14 4 dpi/genotype, kidney: N=4 naïve, N=11-12 4 dpi/genotype). Statistical analysis: **(A–D)** Mann-Whitney test; **(E, F)** 2-way ANOVA.

Given that NKT cells have been shown to play a protective role in the early response to many bacterial infections, we sought to determine whether NKT cells were important for controlling bacterial growth in infected organs after bloodstream challenge with MRSA strain USA300 LAC, an isolate that frequently causes community-acquired skin and soft-tissue infections as well as systemic infections (34). To investigate this, we used C57BL/6 mice (B6) and CD1d^-/-^ mice in the B6 background; CD1d^-/-^ mice do not develop NKT cells due to absence of CD1d expression on double-positive thymocytes, which is required for NKT cell positive selection *in vivo* (28). Once SA enters the bloodstream of mice after infection, its characteristic route of progression through the body is to first be absorbed by liver Kupffer cells within 24 h post infection, then over a period of 2–5 days lyse Kupffer cells and migrate to the kidneys, where it establishes abscesses (35, 36). We thus quantified bacteria in the liver and kidney of SA-infected mice at various times post infection. CD1d^-/-^ mice had increased bacterial burden in the liver and kidney compared to B6 mice at 4 days post infection (dpi), though both groups had equivalent burdens by 8 dpi (**Figures 1A, B**), which suggested that NKT cells were necessary for early control of bacterial growth. This effect was more profound in the liver, where NKT cells were enriched, compared to the kidney. We also compared control of SA systemic infection by NKT cells to another subset of innate-like T cells, mucosal associated invariant T (MAIT) cells, which were activated by vitamin B metabolites present in various bacteria, including SA (37). In contrast to NKT cells, mice lacking MAIT cells (MR1^-/-^) had no discernable difference in bacterial burden in the liver or kidney compared to wild-type littermate controls (MR1^+/+^) at various timepoints (**Figures 1C, D**), highlighting a unique role of NKT cells in the early control of SA infection. In addition, we characterized immune infiltrates in infected kidneys and liver by flow cytometry and identified that neutrophils were increased in the kidneys and liver of CD1d^-/-^ mice relative to B6 mice (**Figures 1E, F**). No significant difference in total number of B cells, macrophages, dendritic cells, and conventional T cells (CD4^+^ and CD8^+^) in the infected liver and kidney were detected between these two groups (**Supplementary Figures 1 and 2**). This data suggested that NKT cells, but not MAIT cells, were critical at early stages of infection to control SA growth and neutrophil infiltration in infected liver and kidneys.

### Type II NKT Cells Were Sufficient to Reduce Bacterial Burden in Infected Liver and Kidneys of Mice Challenged With Sublethal Dose of SA

**Figure 2:**
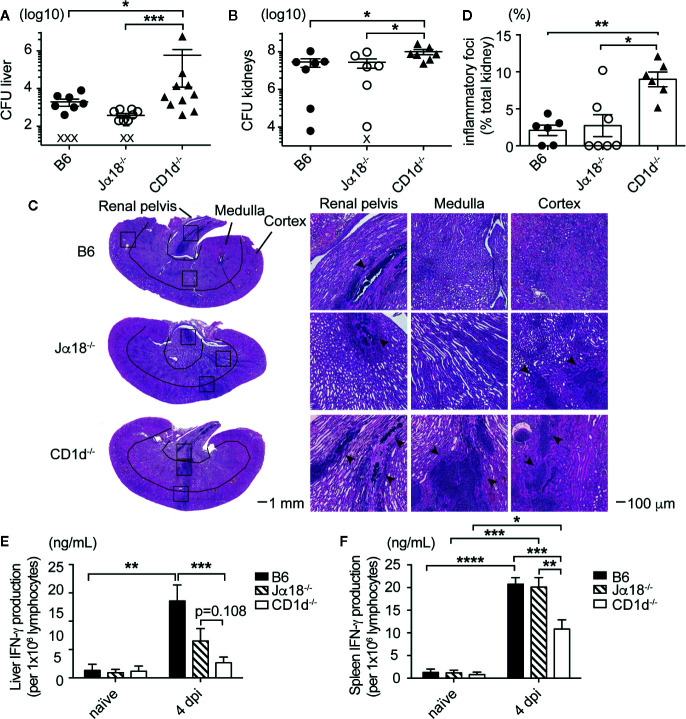
Type II NKT cells are sufficient to control SA growth and mediate cytokine production in infected organs. **(A, B)** CFU quantified in the liver **(A)** and kidney **(B)** of indicated mice at 4 dpi: liver (N=6–13 mice/genotype), kidney (N=6–8 mice/genotype) (X=below limit of detection). **(C, D)** H&E staining of kidney sections at 4 dpi, inflammatory foci area quantified in **(D)**, (N=6-7 mice/genotype). **(E, F)** IFN-γ ELISA of liver **(E)** and spleen **(F)** lymphocytes from 4 dpi mice pulsed with HKSA, unstimulated not graphed (undetectable) (N=2–4 naïve, N=7-10 infected mice/genotype). Statistical analysis: **(A, B, D)** Mann-Whitney test, **(E, F)** 2-way ANOVA. *p < 0.05; **p < 0.01; ***p < 0.001; ****p < 0.0001.

To determine whether type II NKT cells were sufficient to reduce SA bacterial burden, we added the Jα18^-/-^ mouse strain which contains a 10 base pair deletion in the *Traj18* gene and therefore do not develop Vα14-Jα18 expressing iNKT cells, but retained the ability to develop type II NKT cells (27). These mice are an improved version of the original Jα18^-/-^ mice, which have lower TCR diversity due to suppressed transcription of TRAJ gene segments upstream of Traj18 (38). To date, a mouse model lacking type II NKT cells but retaining iNKT cells does not exist, therefore we could not test the efficacy of iNKT cells alone in control of bacterial burdens. B6 (expressing iNKT and II NKT cells) and Jα18^-/-^ mice (expressing only type II NKT cells) had similar bacterial burdens in the liver and kidney at all timepoints tested post infection, while CD1d^-/-^ mice (lacking both NKT cell subsets) again had significantly increased bacterial burdens in the liver and kidney at 4 dpi (**Figures 2A, B**), which suggested that type II NKT cells were sufficient to control SA growth in the absence of iNKT cells at this early timepoint. We also assessed pathology of inflammatory foci and abscess formation in the kidney at 4 dpi by H&E staining (**Figures 2C, D**). Compared to B6 and Jα18^-/-^ mice, where inflammatory foci (marked with arrow heads) were predominantly found in the renal pelvis, CD1d^-/-^ mice had significantly larger inflammatory foci found in all areas of the kidneys (**Figures 2C, D** **, and** **Supplementary Figure 3**). We then determined what cytokines were being induced after SA infection and whether this pattern was altered in mice lacking NKT cells, focusing on the liver and spleen where NKT cells are readily detectable. Liver and spleen lymphocytes from SA-infected CD1d^-/-^ mice produced less IFN-γ in response to *in vitro* restimulation with heat-killed SA (HKSA) compared to infected B6 and Jα18^-/-^ mice at 4 dpi (**Figures 2E, F**). In the liver, iNKT cells were partially responsible for the CD1d-dependent IFN-γ response to HKSA, as Jα18^-/-^ liver lymphocytes exhibited intermediate levels of IFN-γ production between B6 and CD1d^-/-^ mice (**Figure 2E**). However, type II NKT cells were sufficient for CD1d-dependent IFN-γ production in the absence of iNKT cells in the spleen (**Figure 2F**). Together, this data suggested that type II NKT cells were sufficient to reduce bacterial burden and kidney inflammatory infiltrate while enhancing IFN-γ production in the early stages of SA infection.

### NKT Cells Were Activated and Underwent Expansion and Proliferation After SA Infection

**Figure 3:**
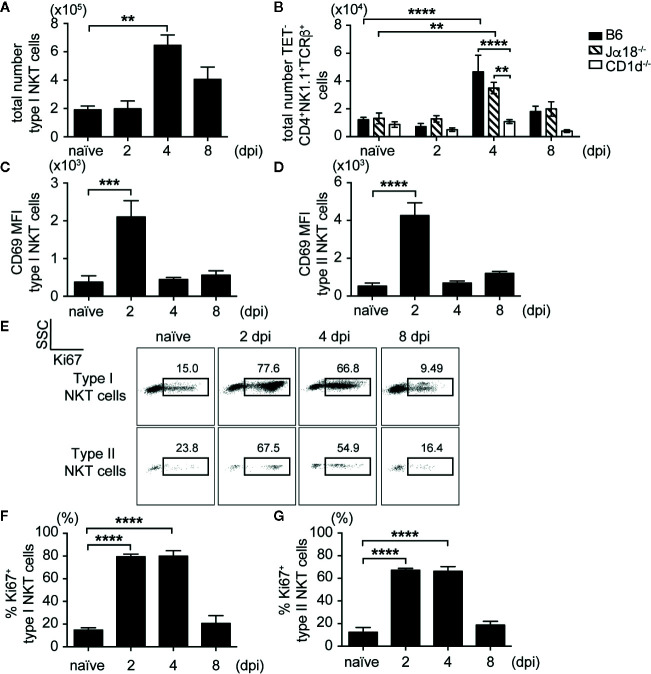
iNKT and type II NKT cells are expanded and activated after SA infection. **(A, B)** Total number of iNKT and type II NKT cells in liver at various times post infection (N=4–5 naïve, N=4–8 infected mice/timepoint). **(C, D)** CD69 mean fluorescence intensity (MFI) of iNKT cells **(C)** and type II NKT cells **(D)** from the liver of SA-infected B6 mice or Jα18^-/-^ mice, respectively (N=4 naïve, N=4–6 infected mice/timepoint). **(E–G)** Ki67 expression in iNKT and type II NKT cells from SA-infected B6 liver. Representative Ki67 dot plot **(E)**, quantified as % of iNKT cells **(F)** and type II NKT cells **(G)** (N=4 naïve, N=3–7 infected mice/timepoint). Statistical analysis: **(A, C–G)** one-way ANOVA, **(B)** 2-way ANOVA. **p < 0.01; ***p < 0.001; ****p < 0.0001.

After showing that mice lacking NKT cells had elevated SA bacterial burden in liver and kidneys, we then determined whether type II NKT cells became activated and expanded during SA infection compared to iNKT cells. We used a CD1d tetramer loaded with PBS57, an α-GalCer analog, to identify iNKT cells *in vivo* (**Supplementary Figure 4A**). Since we did not have specific tetramers to identify type II NKT cells *in vivo*, we used a negative gating strategy to remove iNKT cells and CD8^+^ T cells, then gated on CD4^+^NK1.1^+^TCRβ^+^ cells, which allowed us to look at polyclonal type II NKT cells (**Supplementary Figure 4B**). Both iNKT and type II NKT cells expanded in the liver of SA-infected B6 and Jα18^-/-^ mice (**Figures 3A, B**) at 4 dpi, then contracted by 8 dpi. An increased number of iNKT cells was also detected in the kidney, but not pooled peripheral and kidney draining LN, at 4 dpi (**Supplementary Figures 5B, E**). We confirmed our gating strategy for type II NKT cells using CD1d^-/-^ mice; most of the TET^-^CD8α^-^CD4^+^NK1.1^+^TCRβ^+^ cells were CD1d-restricted as the percentage and total number of this population was reduced in CD1d^-/-^ mice after infection (**Supplementary Figure 4B** **and** **Figure 3B**). Due to the small number of type II NKT cells in the mouse relative to iNKT cells, we were only able to detect CD1d-restricted type II NKT cells in the liver (**Supplementary Figure 4B**) where they were enriched. At 2 dpi, both iNKT and type II NKT cells in the liver already upregulated activation marker CD69 (**Figures 3C, D**). In addition, significant proliferation of both iNKT and type II NKT cells from SA-infected mice was detected at 2 and 4 dpi (**Figures 3E–G**), as reflected by the increased percentage of Ki67^+^ cells compared to naïve mice. By 8 dpi, Ki67 expression was reduced to naïve levels, suggesting that these cells underwent contraction or cell death after their function was performed. This data demonstrated that both iNKT and type II NKT cells were poised to respond quickly to SA infection as denoted by upregulation of activation markers and cell expansion at early times post-infection.

As previously described, type II NKT cells encompass polyclonal CD1d-restricted T cells recognizing lipid antigens from diverse bacterial species (21, 22). To determine whether specific type II NKT cell populations were expanding after infection, we probed type II NKT cell surface expression of specific Vβ chains in naïve and infected Jα18^-/-^ mice. Similar to previously published work (39), the majority of polyclonal type II NKT cells utilized the Vβ8.1/8.2 chain, while smaller populations expressed diverse Vβ chains (**Supplementary Figure 6**). We saw no significant alteration of Vβ chain usage in naïve vs. infected mice, which suggested that type II NKT cells were polyclonally expanding after infection.

### NKT Cells Produced SA-Specific IFN-γ After Infection

**Figure 4:**
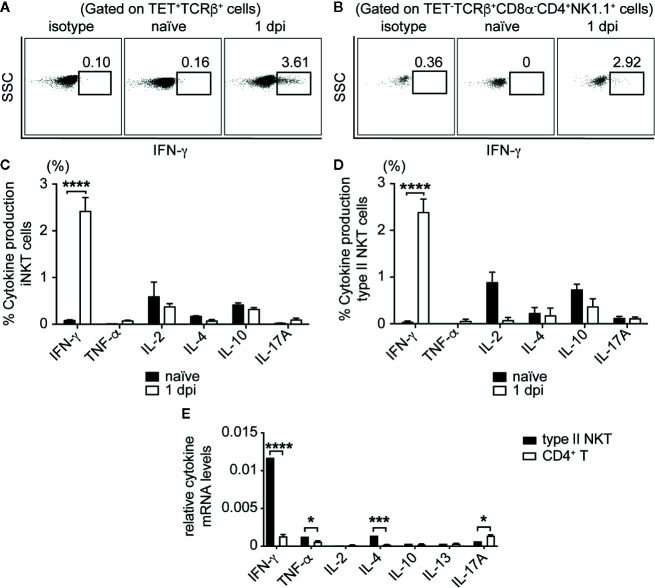
iNKT and type II NKT cells produce IFN-γ after SA infection. **(A, B)** Representative FACS plots of IFN-γ production of iNKT **(A)** and type II NKT **(B)** cells at 1 dpi from B6 liver, cultured directly *ex vivo*. **(C, D)** Quantified % cytokine production, gated on iNKT cells **(C)** and type II NKT cells **(D)**. (N=3–6 naïve, N=5–12 infected mice). **(E)** rtPCR of cytokine mRNA of type II NKT cells sorted from 4 dpi Jα18^-/-^ mouse pooled liver lymphocytes (N=10 mice) compared to sorted conventional CD4^+^NK1.1^-^ T cells from Jα18^-/-^ splenocytes (N=2 mice), data represented as cytokine mRNA levels relative to β-actin. Statistical analysis: **(C–E)** 2-way ANOVA. *p < 0.05; ***p < 0.001; ****p < 0.0001.

Having demonstrated that NKT cells were activated during SA infection, we next determined whether NKT cells were producing cytokines directly in response to infection that could be driving the reduction in CFU we saw in the early phase of infection (**Figures 1** and **2**). To this end, we isolated liver lymphocytes from SA-infected B6 mice at various times post-infection and performed intracellular staining *ex vivo* without stimulation. Both iNKT and type II NKT cells produced IFN-γ in the liver at 1 dpi in infected B6 mice, but not in naïve controls (**Figures 4A, B**). The production of IL-2, IL-4, IL-10, IL-17A, and TNF-α by NKT cells were either absent or remained unchanged between naïve and infected mice (**Figures 4C, D**). No cytokine production was detected directly *ex vivo* at later times post infection, which suggested that NKT cells produced a burst of IFN-γ during the initial phase of infection but not at later times. To determine whether type II NKT cells had upregulated cytokine mRNA relative to conventional T cells after infection, we isolated RNA from sorted type II NKT cells at 4 dpi from pooled liver lymphocytes of Jα18^-/-^ mice for qPCR. We focused on this timepoint because this was the point at which we saw the greatest expansion of type II NKT cells and were able to sort enough cells to obtain RNA (**Figure 3B**). RNA isolated from conventional CD4^+^NK1.1^-^ T cells from the spleen of these mice was used for comparison purpose. At 4 dpi, type II NKT cells had upregulated Th1 and Th2 cytokine mRNA relative to conventional CD4^+^ T cells (**Figure 4E**), suggesting that type II NKT cells were poised to respond to SA at an earlier timepoint than conventional T cells.

Systemic exposure to a number of microorganisms has been shown to render iNKT cells hyporesponsive to antigen restimulation (40–42). Indeed, liver iNKT cells from SA-infected WT mice produced less cytokines, including IFN-γ, IL-4, IL-13, and GM-CSF, in response to *in vitro* α-GalCer stimulation compared to iNKT cells from naïve mice (**Supplementary Figures 7A, B**). In addition, iNKT cells from infected mice produced less IFN-γ compared to their naïve counterparts in response to PMA/Ionomycin (**Supplementary Figures 7C, D**). These data demonstrated that iNKT cells were functionally anergic to restimulation after SA infection.

### Type II NKT Cell Required Both TCR-Dependent and Independent Signals to Become Activated and Produce IFN-γ in Response to SA Lipid Antigens

**Figure 5:**
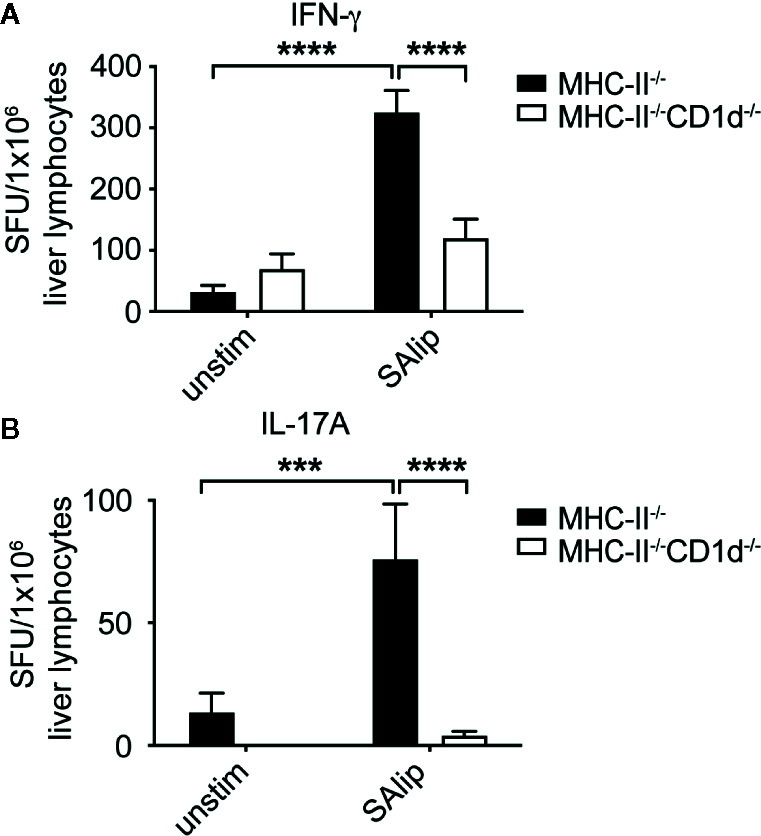
Type II NKT cells require CD1d to recognize total SA lipids. **(A, B)** IFN-γ **(A)** and IL-17A **(B)** ELISPOT of Jα18^-/-^ total liver lymphocytes collected from 4 dpi livers and co-cultured with MHC-II^-/-^ or MHC-II^-/-^CD1d^-/-^ DCs, unpulsed or pulsed overnight with 10 µg/ml total SA lipids (SAlip) (N=4 mice/experiment, representative 1 of 3). Statistical analysis: **(A, B)** 2-way ANOVA.***p < 0.001; ****p < 0.0001.

Given that iNKT cells were dispensable for the control of bacterial burden in systemic SA infection, we focused on how type II NKT cells were activated and contributed to protection. Work from our lab demonstrated that group 1 CD1-restricted T cells recognized polar lipids derived from the cell membrane of SA (30), but no SA lipid antigen recognition by type II NKT cells has been described to date. To determine whether type II NKT cells can recognize SA lipids, total liver lymphocytes from infected Jα18^-/-^ mice were co-cultured with BMDCs unpulsed or pulsed with total SA lipids. We utilized MHC class II knockout (MHC-II^-/-^) DCs to remove any MHC-II conventional CD4 T cell-mediated response and compared MHC-II^-/-^ to MHC-II^-/-^CD1d^-/-^ conditions to determine whether cytokine production to total SA lipids was CD1d-restricted. Total SA lipid pulsed MHCII^-/-^ DCs induced CD1d-restrcited IFN-γ and IL-17A production by liver lymphocytes isolated from Jα18^-/-^ mice at 4 dpi (**Figures 5A, B**).

**Figure 6:**
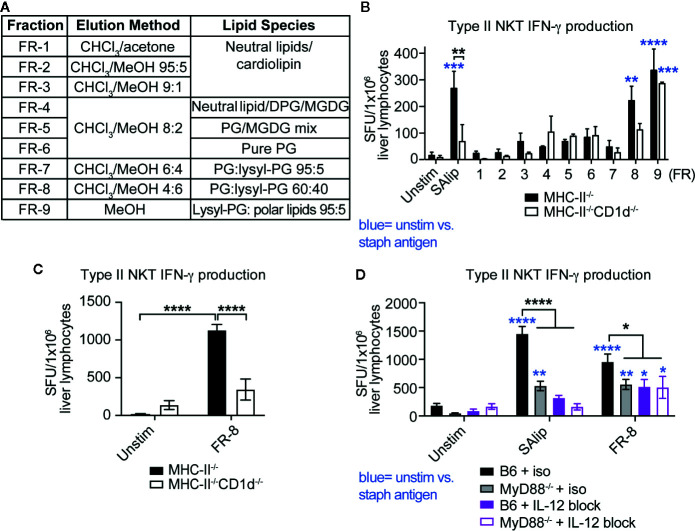
Type II NKT cells produce MyD88-dependent CD1d-restricted IFN-γ to polar SA lipids. **(A)** List of SA lipid fractions, isolated from total SA lipids using silica gel column chromatography and chloroform-methanol gradient in order of increasing polarity, PG=phosphatidylglycerol, DPG= diacylphosphatidylglycerol, MGDG= monogalactosyldiacylglycerol. **(B–D)** ELISPOT of IFN-γ production by T cells enriched from 4 dpi Jα18^-/-^ liver lymphocytes: **(B, C)** MHC-II^-/-^ and MHC-II^-/-^CD1d^-/-^ DCs or **(D)** B6 and MyD88^-/-^ DCs +/- IL-12 blocking antibody pulsed with total SA lipids and total SA lipid fractions (FR-1 to FR-9, 10 µg/ml pulsed overnight). **(B)** N=5–7 pooled mice, 2/3 replicates per condition, representative of 2 experiments. Statistical analysis: **(B–D)** 2-way ANOVA. *p < 0.05; **p < 0.01; ***p < 0.001; ****p < 0.0001.

To determine whether specific lipids from SA induced IFN-γ production by type II NKT cells, we used a chloroform (ChCl_3_)-methanol (MeOH) step gradient to fractionate total SA lipids into nine fractions based on polarity and identified dominant lipid species in each fraction by thin-layer chromatography (TLC) as described in (30) (**Figure 6A**). Type II NKT cells were enriched from livers of infected Jα18^-/-^ mice by depletion of CD8^+^ T cells and various APCs, then co-cultured with MHC-II^-/-^ and MHC-II^-/-^CD1d^-/-^ DCs pulsed with total SA lipids and SA lipid fractions. Previous studies using type II NKT cell hybridomas identified PG and cardiolipin species from *Mtb* and *C.* *glutamicum* that induced CD1d-restricted cytokine production (21). Our screening identified that fractions FR-8 and FR-9, containing both PG and lysyl-phosphatidylglycerol (lysyl-PG) species, induced IFN-γ production by enriched type II NKT cells (**Figure 6B**). Only FR-8, containing a ratio of 60:40 PG: lysyl-PG, showed a trend for CD1d-restriction, whereas FR-9 induced IFN-γ production completely independent of CD1d. To confirm whether FR-8 induced IFN-γ production was CD1d-restricted, we further enriched type II NKT cells from Jα18^-/-^ liver lymphocytes, by additional depletion of γδ T cells and performed IFN-γ ELISPOT with MHC-II^-/-^ and MHC-II^-/-^CD1d^-/-^ DCs pulsed with FR-8. We showed that FR-8 induced CD1d-restricted IFN-γ production by type II NKT cells (**Figure 6C**). In addition to TCR-CD1d mediated antigen-specific activation, iNKT cells were shown to be activated by MyD88-dependent signals, which required IL-12 and IL-18 production by TLR activated APCs (43). To determine whether IFN-γ production by type II NKT cells in response to SA lipids was MyD88 and/or cytokine-dependent, we used B6 and MyD88^-/-^ DCs pulsed with total SA lipids or FR-8 in the presence of IL-12 blocking antibody (**Figure 6D**). Total SA lipid and FR-8 induced IFN-γ production was dependent on both MyD88 and IL-12 production, suggesting both CD1d-TCR engagement and IL-12 producing APCs were required for optimal IFN-γ production by type II NKT cells in response to phospholipids from SA. In summary, polyclonal type II NKT cells required CD1d, MyD88, and IL-12 for IFN-γ production to be activated by polar lipid species from the cell membrane of SA.

### Adoptive Transfer of Type II NKT Cells Protected Mice From Systemic SA Infection

**Figure 7:**
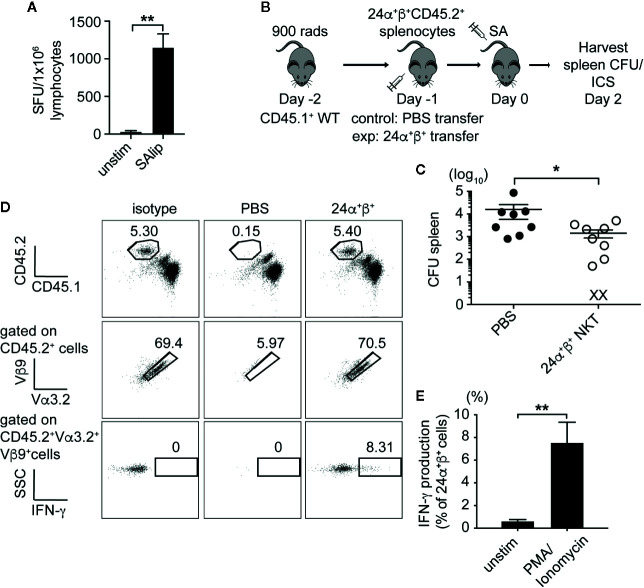
Type II NKT cells protect mice from systemic SA infection. **(A)** IFN-γ ELISPOT of naïve 24α^+^β^+^ lymphocytes from liver co-cultured with B6 DCs unpulsed or pulsed with total SA lipids (SAlip) (10 µg/ml). **(B)** Schematic of type II NKT cell adoptive transfer experiment. **(C)** Bacterial CFU in the spleen of recipient mice at 2 dpi, PBS= control group **(D, E)**. Representative FACS plots of adoptively transferred 24α^+^β^+^ NKT cells from spleen of 2 dpi recipient, % IFN-γ production after stimulation with PMA/Ionomycin or unstimulated (2 h + 4 h BFA), quantified in E (N=8–10 mice/group). Statistical analysis: **(A)** one-way ANOVA, **(C)** Mann-Whitney test, **(E)** 2-way ANOVA. *p < 0.05; **p < 0.01.

Given the low prevalence of type II NKT cells in wild-type mice, we decided to use T cells from a well-characterized 24αβ TCR transgenic mice expressing the Vα3.2^+^Vβ9^+^ TCR from a type II NKT cell hybridoma (9, 29) to test for the ability of type II NKT cells to protect against SA infection. Similar to polyclonal type II NKT cells, 24αβ Tg T cells produced IFN-γ in response to total SA lipids (**Figure 7A**). To determine if 24αβ Tg T cells protected mice from SA infection, we set up an adoptive transfer experiment using congenic markers to track 24αβ Tg T cells *in vivo* after infection (**Figure 7B**). A substantial number of 24αβTg T cells were detected in the spleen of recipient mice, with very few cells identified in the liver (**Figure 7D**, **Supplementary Figure 8**). As a result, we observed a significant reduction in CFU at 2 dpi in the spleen of mice receiving 24αβ Tg T cells compared to control (PBS injected) mice (**Figure 7C**), demonstrating that type II NKT cells protected mice from SA infection by contributing to reduced CFU. The 24αβ Tg T cells isolated from SA-infected recipient mice produced significant amounts of IFN-γ after *ex vivo* stimulation with PMA/Ionomycin (**Figures 7D, E**). This data demonstrated that type II NKT cells contributed to reduction in bacterial burden after SA infection and this effect was likely through enhanced IFN-γ production.

### Type II NKT Cells Expanded in SA Bacteremic Mice and Human Patients

**Figure 8:**
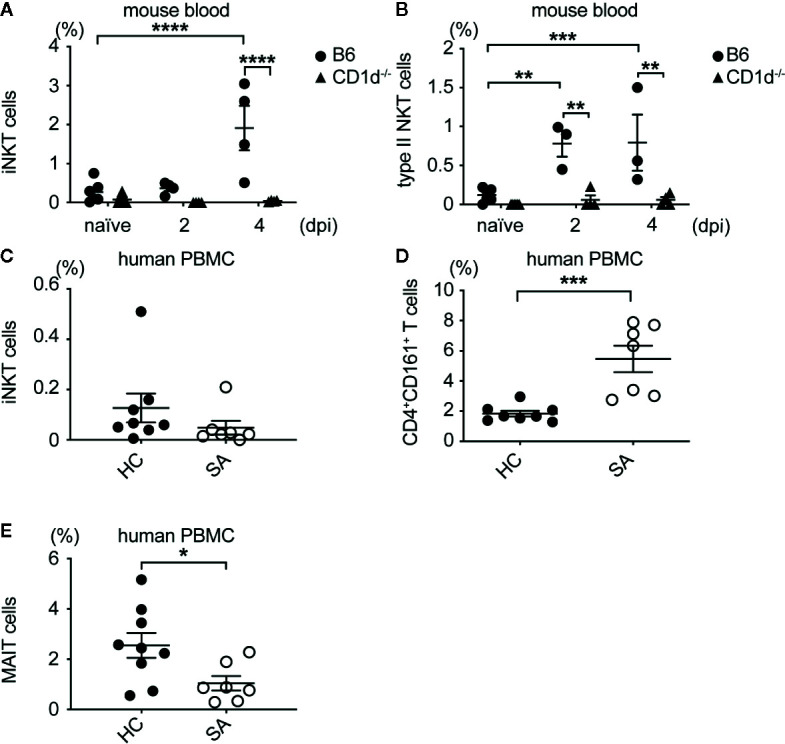
Type II NKT cells expanded in SA bacteremic mice and human patients. **(A)** Percentage of iNKT cells in blood of B6 and CD1d^-/-^ mice. **(B)** Percentage of type II NKT cells in blood of B6 and CD1d^-/-^ mice, **(A, B)** N=7 naïve, three to four infected mice/genotype. **(C)** Percentage of iNKT cells in SA infected (SA) and healthy control (21) PBMC. **(D)** Percentage of type II NKT cells in SA and HC PBMC. **(E)** Percentage of MAIT cells in SA and HC PBMC, **(C–E)** N=8–9 HC, seven SA patients. Statistical analysis: **(A–C, E)** 2-way ANOVA, **(D)** student’s t-test. *p < 0.05; **p < 0.01; ***p < 0.001; ****p < 0.0001.

To determine whether NKT cells expanded in the blood of patients with SA bacteremia, we compared frequency of iNKT and type II NKT cells in peripheral blood mononuclear cells (PBMC) isolated from patients at Northwestern Memorial Hospital with methicillin-sensitive or methicillin-resistant SA bacteremia to that from healthy donors. While iNKT cells were increased in the blood of mice infected with SA, iNKT cells (defined as hCD1d/PBS57 tetramer^+^CD3^+^ cells) were decreased in SA infected patients compared to healthy controls, although this was not statistically significant (**Figures 8A, C**). Type II NKT cells were increased in the blood of mice with SA bacteremia (**Figure 8B**). Similarly, SA bacteremic patients had statistically higher percentage of CD4^+^CD161^+^hCD1d/PBS57 tetramer^-^ T cells, enriched for type II NKT cells, compared to healthy individuals (**Figure 8D**). Since a substantial proportion of CD161^+^ T cells in human PBMC are MAIT cells (defined as MR1/5-OP-RU tetramer^+^ CD3^+^ cells), we also determined the frequency of MAIT cells in SA bacteremic patients and healthy controls. In contrast to CD4^+^CD161^+^ T cells, the percentage of MAIT cells was decreased in SA bacteremic patients compared to healthy controls (**Figure 8E**). In addition, most of the MAIT cells in SA-infected individuals were DN or CD8^+^ cells. Thus, MAIT cells were unlikely to account for the increased CD4^+^CD161^+^ T cell population observed in SA-infected individuals. In conclusion, SA bacteremic patients had increased frequency of CD4^+^CD161^+^ T cells compared to healthy controls suggesting that type II NKT cells could be relevant in the human setting for combatting SA bacteremia.

## Discussion

The ability of NKT cells to become activated and produce cytokine early in response to infection has made these cell populations key players in control of many bacterial infections. Here we showed that NKT cells were activated and proliferating in response to SA bacteremia. Though both iNKT and type II NKT cells were activated during SA infection, only type II NKT cells were necessary for reducing bacterial burden and neutrophilic infiltrate into liver and kidneys. Type II NKT cells recognized polar lipids derived from the cell membrane of SA, with IFN-γ production dependent on both TCR-CD1d engagement and TLR-mediated activation. Using monoclonal type II NKT cells from TCR transgenic mice, we showed that adoptive transfer of type II NKT cells lowered bacterial burden of SA in the spleen, which was correlated to IFN-γ production. Lastly, we demonstrated the clinical relevance of our findings by showing that a subset of T cells, which included type II NKT cells, was expanded in the blood of SA bacteremic patients versus healthy controls.

Previous studies demonstrated that NKT cells were not required for increased survival during SA lethal challenge (20, 44). Using sublethal dose of systemic SA infection, we observed a role for NKT cells in controlling liver and kidney CFU (**Figures 1A, B** and **2A, B**). While MAIT cells have been implicated in control of *Francisella tularensis* pneumonia, *E. coli* systemic infection, and *Legionella longbeacha*e pneumonia, their role in SA infection has not previously been explored to our knowledge (37, 45, 46). We showed that mice lacking MAIT cells, which have similar activation kinetics to NKT cells, did not have an increase in bacterial burden after SA infection, suggesting that NKT cell control of SA bacterial burden was unique (**Figures 1C, D**). It is possible that MAIT cells may play a role in SA pneumonia considering that MAIT cells are enriched in the lung and SA harbors the necessary proteins to produce riboflavin metabolites recognized by MAIT cells, however this has yet to be studied. CD1d^-/-^ kidneys had larger areas of inflammatory foci, composed of majority infiltrating neutrophils with smaller amounts of macrophage, DC, and T cell infiltrate (**Figures 1E, F**, **Figures 2C, D**, **Supplementary Figures S1** and **S2**). Loss of NKT cells also led to decreased IFN-γ production by total liver and spleen lymphocytes in response to HKSA (**Figures 2E, F**), suggesting that IFN-γ was beneficial in limiting growth of SA at this early timepoint. Indeed, IFN-γ production by iNKT cells was demonstrated to enhance innate protection against murine *L. monocytogenes* infection (47).

Similar to a previous study looking at iNKT cells in SA infection (20), iNKT cells became activated and proliferated during the early stage of SA systemic infection (**Figure 3**). It is important to note that this study utilized a methicillin-sensitive strain of SA (LS-1), which contains superantigens different from methicillin-resistant USA300 SA strain. SA superantigen SEB has been show to activate iNKT cells independent of specific antigen, however USA300 SA strain used in our study does not contain dominant superantigens such as SEB (48–50). Due to the availability of a tetramer to identify iNKT cells *in vivo*, this population of NKT cells has been much more widely studied in bacterial infections compared to type II NKT cells. Though iNKT cells were previously shown to be activated during SA infection, this is the first study to our knowledge demonstrating that polyclonal type II NKT cells undergo activation and expansion during SA infection. A mouse study that explored the role of type II NKT cells in SA-induced sepsis showed that immunization of Jα18^-/-^ and CD1d^-/-^ mice with the type II NKT cell specific self-lipid sulfatide conferred CD1d-restricted protection, however they did not track type II NKT cells *in vivo* (20). We have previously used IL-4 reporter mice to track polyclonal type II NKT cells in naive mice (39). In initial experiments, we had attempted to use IL-4 reporter mice on the Ja18^-/-^ background to track type II NKT cells *in vivo*, however, we did not see a significant expansion of this population after SA infection, despite the presence of low levels of IL-4 mRNA in sorted type II NKT cells (**Figure 4E**). This could be due to the skewing of the NKT cell response after SA infection to IFN-γ production. Since a tetramer is not available to study type II NKT cells *in vivo*, we used surface marker expression and CD1d^-/-^ mice as negative controls to identify polyclonal type II NKT cells. We confirmed that the majority of CD4^+^NK1.1^+^ T cells were truly type II NKT cells, since this population was reduced in CD1d^-/-^ mice and did not expand in CD1d^-/-^ mice relative to B6 and Jα18^-/-^ mice after SA infection (**Supplementary Figure 4** and **Figure 3B**). Additional experiments using SA lipid antigens functionally demonstrated that this population was CD1d-restricted (**Figures 5** and **6**). In addition to characterizing polyclonal type II NKT cell activation and expansion (**Figures 3B, D, G**), we determined that these cells utilized diverse Vβ chains, which were polyclonally expanded, rather than preferentially expanded for a single clone, during SA infection (**Supplementary Figure 6**).

iNKT cells have been shown to increase IFN-γ production in direct response to other bacterial infections, including *S. pneumoniae*, *L. monocytogenes*, and *S. typhimurium* (47, 51, 52), but this was the first study to our knowledge demonstrating that type II NKT cells upregulated IFN-γ production in direct response to SA infection *in vivo*. This is in keeping with the pre-activated state of NKT cells, which encode cytokine mRNA in their cytoplasm that can be quickly transcribed into cytokine proteins and secreted within minutes to hours after encounter of stimuli (53). Indeed, when we sorted type II NKT cells from SA infected mice and compared their cytokine mRNA levels to conventional CD4^+^ T cells, type II NKT cells had upregulated IFN-γ and IL-4 mRNA compared to conventional CD4^+^ T cells (**Figure 4E**). While both iNKT and type II NKT cells produced IFN-γ in response to SA infection, iNKT cells became hyporesponsive to restimulation with α-GalCer after infection (**Supplementary Figures 7A, B**). A similar hyporesponsive phenotype to restimulation with α-GalCer has been observed with mice infected with *L. monocytogenes*, and *M. bovis* infection (40, 41). Additionally, iNKT cells from mice injected with heat-killed SA, *E. coli*, or *S. typhimurium* had a reduced capacity to produce cytokines to α-GalCer *ex vivo* stimulation, even up to 3 weeks post killed-bacteria injection (42). Whereas bypassing of proximal TCR signaling with PMA/Ionomycin was able to rescue hyporesponsiveness to α-GalCer after heat-killed *E. coli* injection, iNKT cells from SA infected mice were unable to produce naïve levels of IFN-γ after PMA/Ionomycin stimulation, suggesting an intrinsic defect in these cells to produce cytokines after SA infection (**Supplementary Figures 7C, D**) and (42). iNKT cell activation can lead to detrimental immune pathology in atherosclerosis and alcoholic liver disease (54, 55). It is possible that the induction of a hyporesponsive phenotype in iNKT cells after SA infection was a protective mechanism induced by the host to reduce overactivation of expanded iNKT cells, as has been suggested in other bacterial infections. This data demonstrated that type II NKT cells but not iNKT cells played the dominant role during SA infection.

The SA cell membrane is composed primarily of PG, lysyl-PG, and cardiolipin lipid species (56). We showed here that a mixture of polar lipids (FR-8) induced IFN-γ production by type II NKT cells that was both CD1d-restricted and IL-12-dependent (**Figure 5** and **Figures 6C, D**). In contrast, the fraction containing pure PG (FR-6) did not induce any IFN-γ production and the fraction containing 95% lysyl-PG + 5% unidentified polar lipids (FR-9) induced IFN-γ production that was not CD1d-restricted. It is important to note that SA contains multiple PG and lysyl-PG species of varying chain lengths. A study using HPLC coupled with mass spectrometry (LC-Q-TOF-MS) to characterize the SA lipidome identified multiple species of PG and lysyl-PG of varying chain lengths eluting at different times during fractionation, with PG species being enriched in methicillin-resistant SA compared to methicillin-sensitive SA strains (57). Fatty acid chain length and confirmation of fatty acid chains in the cis or trans position affects lipid loading onto CD1d molecules and subsequent NKT cell TCR engagement (58–60). PGs of different chain lengths have been identified from *Mtb*, *C. glutamicum*, and *L. monocytogenes* with the ability to stimulate type II NKT cell hybridomas; the efficiency of activation was dependent on the affinity of biding to CD1d (21, 22). Therefore, it was possible that PG/lysyl-PG lipids from FR-8 contained unique fatty acid chain lengths that affected the binding affinity of these lipids to CD1d and the strength of type II NKT cell TCR engagement, relative to PG from FR-6 and lysyl-PG species from FR-9. Further work needs to be done to characterize the type II NKT cell response to SA lipids, including isolating individual SA lysyl-PG or PG lipid moieties for further characterization. Previous work from our lab showed that PG from FR-8 induced group 1 CD1b/c-restricted IL-17A and IFN-γ production in the lymph nodes of group 1 CD1 transgenic mice (hCD1Tg) (30). This suggested that FR-8 contained lipid species which stimulated both group 1 CD1-restricted T cells and type II NKT cells.

Given that type II NKT cells were necessary for reducing bacterial burden and neutrophil infiltration at an early time post SA infection, we sought to determine mechanistically how type II NKT cells provided protection *in vivo*. Since polyclonal type II NKT cells were too few in the mouse to isolate and adoptively transfer directly, we used a well characterized type II NKT cell transgenic mouse, 24αβ Tg, to perform adoptive transfer experiments. 24αβ cells were shown to play both beneficial and detrimental roles during models of inflammation and autoimmunity. 24αβ cells suppressed type I diabetes onset in mice, but exacerbated colitis when CD1d was overexpressed in 24αβ CD1dTg^+^ mice (61, 62). Additionally, the VIII24 hybridoma, the type II NKT cell clone used to make 24αβ Tg mice, recognized both polar *Mtb* lipids and tumor-derived self-lipids (21, 63). We showed that 24αβ cells produced IFN-γ in response to total SA lipids (**Figure 7A**). Irradiated CD45.1^+^ mice that received splenic 24αβ cells showed enhanced recruitment of 24αβ cells to the spleen of recipient mice and reduced bacterial burden in the spleen after SA infection, demonstrating a direct role for type II NKT cells in control of SA bacterial burden (**Figure 7C**, **Supplementary Figure 8**). 24αβ cells produced IFN-γ after adoptive transfer, similar to polyclonal type II NKT cells after infection (**Figures 7D, E**). A recent study showed that 24αβ cells stimulated with TLR agonists in the presence of DCs produced IFN-γ independent of TCR receptor signaling and dependent on IL-12 (64). While it is unclear from our study whether TCR-CD1d or IL-12 played the dominant role in 24αβ cell protection *in vivo*, our data in the polyclonal setting suggested that both TCR-CD1d signaling and IL-12 were required for IFN-γ production to SA lipid antigens *ex vivo* (**Figure 6**). Indeed, it has been suggested that during an infection, these pathways could be acting simultaneously to activate type II NKT cells (19). It will be important to repeat these experiments with SA lipid antigen-specific type II NKT cell clones to determine whether antigen-specific type II NKT cells directly contribute to control of infection.

Lastly, we demonstrated that a population of CD161^+^CD4^+^ T cells, which includes type II NKT cells, were expanded in human SA bacteremic patients and mice (**Figures 8B–D**). Type II NKT cells are more enriched in human PBMC compared to iNKT cells, therefore we expected this population to make up a majority of the NKT cell pool in our healthy control patients. RNAseq of sorted CD161^+^ T cells from human PBMC identified that this population shared conserved transcripts found in type II NKT cells, γδ T cells, and conventional Th17 producing CD4^+^ T cells (65). Within this group, the majority of human γδ T cells are CD8^+^ or DN, with CD4^+^ γδ T cells being rare (66), therefore it is unlikely that CD161^+^CD4^+^ T cells are γδ T cells, and rather this population is enriched for type II NKT cells and Th17 cells. In contrast to type II NKT cells, iNKT cells were reduced in the blood of active *Mtb* infected patients vs latent infected patients (32). We also saw a decrease in iNKT cells in SA infected patients vs. healthy controls (**Figure 8C**), while in mice iNKT cells were increased in the blood after infection (**Figure 8A**). Further studies are needed to determine if iNKT cells are being recruited to infected tissue in humans, and whether they have any benefit to the outcome of SA infection. The percentage of MAIT cells was decreased in SA bacteremic patients, therefore the CD161^+^CD4^+^ T cell increase was unlikely due to MAIT cell expansion (**Figure 8E**). Functional assays with enriched T cells from SA patients will need to be performed to confirm that human CD161^+^CD4^+^ T cells are CD1d-restricted and whether they produce cytokine to SA lipid antigens in healthy vs. SA bacteremic patients.

We showed in this work that type II NKT cells, a T cell population that is understudied relative to iNKT cells, played a role in protection against SA infection. Due to the lack of specific markers to identify type II NKT cells *in vivo*, we were limited in our ability to study this cell population and resorted to the use of genetic knockout mice to track their expansion and activation after SA infection. Previous studies have used CD1d tetramers loaded with lipid antigens such as sulfatide to track subsets of type II NKT cells *in vivo* (67). We identified a SA lipid fraction containing PG: lysyl-PG polar lipids that were recognized by type II NKT cells in a CD1d-dependent manner, however further work needs to be done to identify the specific lipid within this fraction that is recognized by type II NKT cells. Once identified, this lipid could be loaded onto a CD1d tetramer to track type II NKT cells *in vivo* during infection. Similarly, in human PBMC we were limited in our ability to identify type II NKT cells and resorted to a gating strategy that has previously been identified to include type II NKT cells (65). Due to the presence of Th17 cells within this population, we cannot definitively say that these cells are type II NKT cells but refer to them as NKT-like cells. Functional assays using our SA lipid antigens will need to be performed to confirm that this population is CD1d-restricted.

In conclusion, we showed that type II NKT cells and not iNKT cells, were important for reducing bacterial burden and controlling neutrophil infiltrate to infected liver and kidneys after SA systemic infection. This protection was mediated by type II NKT cell IFN-γ production, which required both CD1d-TCR engagement and IL-12 production/MyD88 engagement by APCs. We also determined that PG and lysyl-PG species from the cell membrane of SA induced type II NKT cell cytokine production. Because of the nonpolymorphic nature of CD1d molecules, targeting of type II NKT cells with a lipid antigen vaccine could be a novel method to induce protection against SA infections where previous vaccines have failed.

## Data Availability Statement

The raw data supporting the conclusions of this article will be made available by the authors, without undue reservation.

## Ethics Statement

The studies involving human participants were reviewed and approved by the protocol was approved by the Northwestern Institutional Review Board (IRB #STU0001210512). The patients/participants provided their written informed consent to participate in this study. The animal study was reviewed and approved by The protocol was approved by the Animal Care and Use Committee of the Northwestern University (Protocol number: IS00001659).

## Author Contributions

SG, LV, LC, EM, YC, and EB performed the experiments. SG, CQ, Y-HC, LG, and C-RW designed the experiments. SG, LV, and C-RW analyzed the data. SG, LV, and C-RW prepared the manuscript. All authors contributed to the article and approved the submitted version.

## Funding

This work was supported by NIH grants R01 AI43407 and R01 AI057460 to C-RW.

## Conflict of Interest

The authors declare that the research was conducted in the absence of any commercial or financial relationships that could be construed as a potential conflict of interest.
