# Supplementary material for: Type II Natural Killer T Cells Contribute to Protection Against Systemic Methicillin-Resistant Staphylococcus aureus Infection
Source: Front Immunol. 2020 Nov 18;11:610010. doi: 10.3389/fimmu.2020.610010 (PMC7708336; doi:10.3389/fimmu.2020.610010)
Supplement: Supplementary file 1 [file DataSheet_1.pdf]

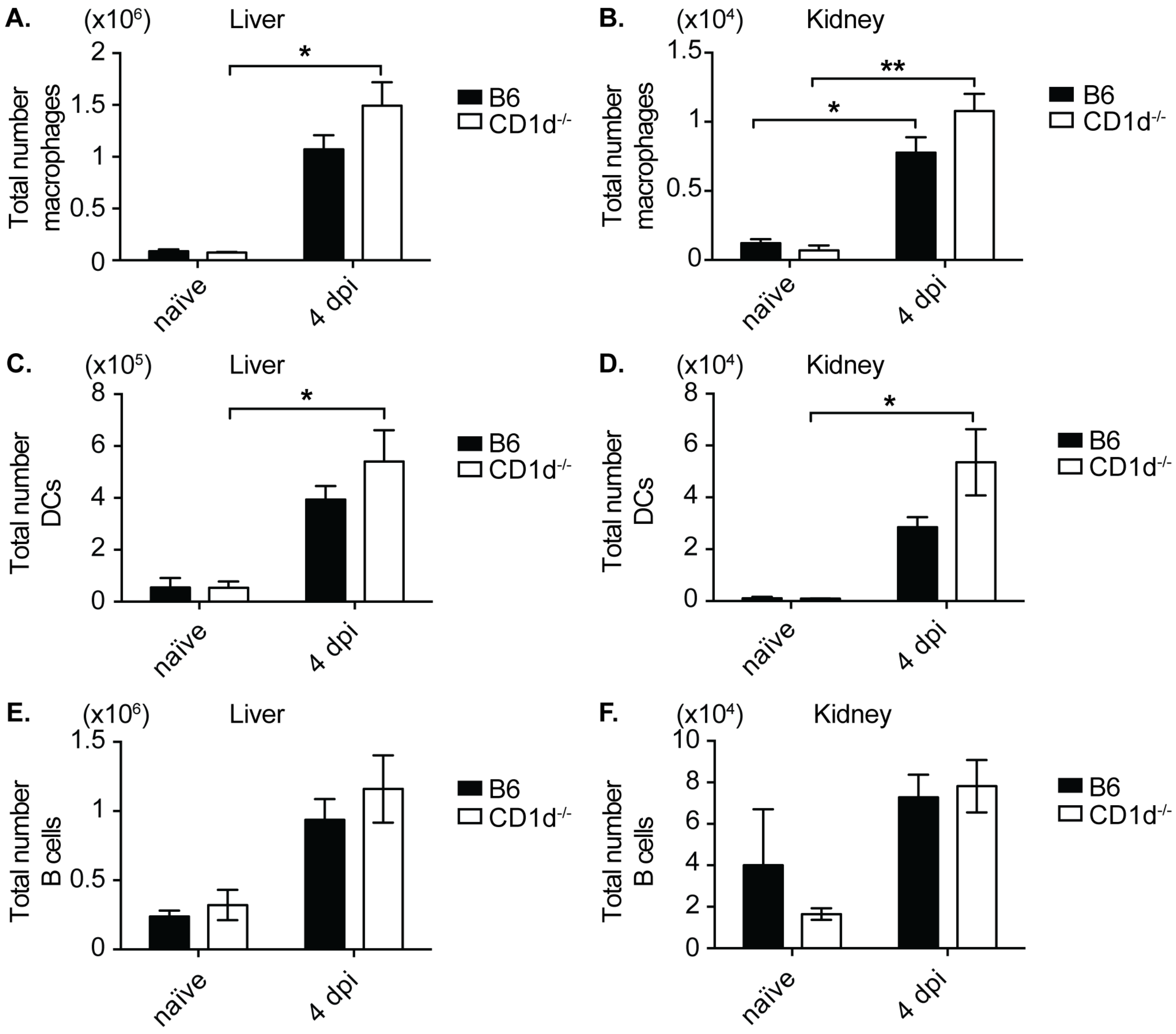

S1 Fig. Absence of NKT cells does not alter other antigen presenting cell expansion in infected organs.

(A, B) Total number of macrophages in liver (A) and kidney (B) at 4 dpi. **(C, D)** Total number of dendritic cells (DC) in liver (C) and kidney (D) at 4 dpi. **(E, F)** Total number of B cells in liver (E) and kidney (F) at 4 dpi. Kidney (N=2 naïve, N=7 4 dpi mice), liver (N=3 naïve, N=9-10 4 dpi mice). Statistical analysis: (A-F) 2-way ANOVA.

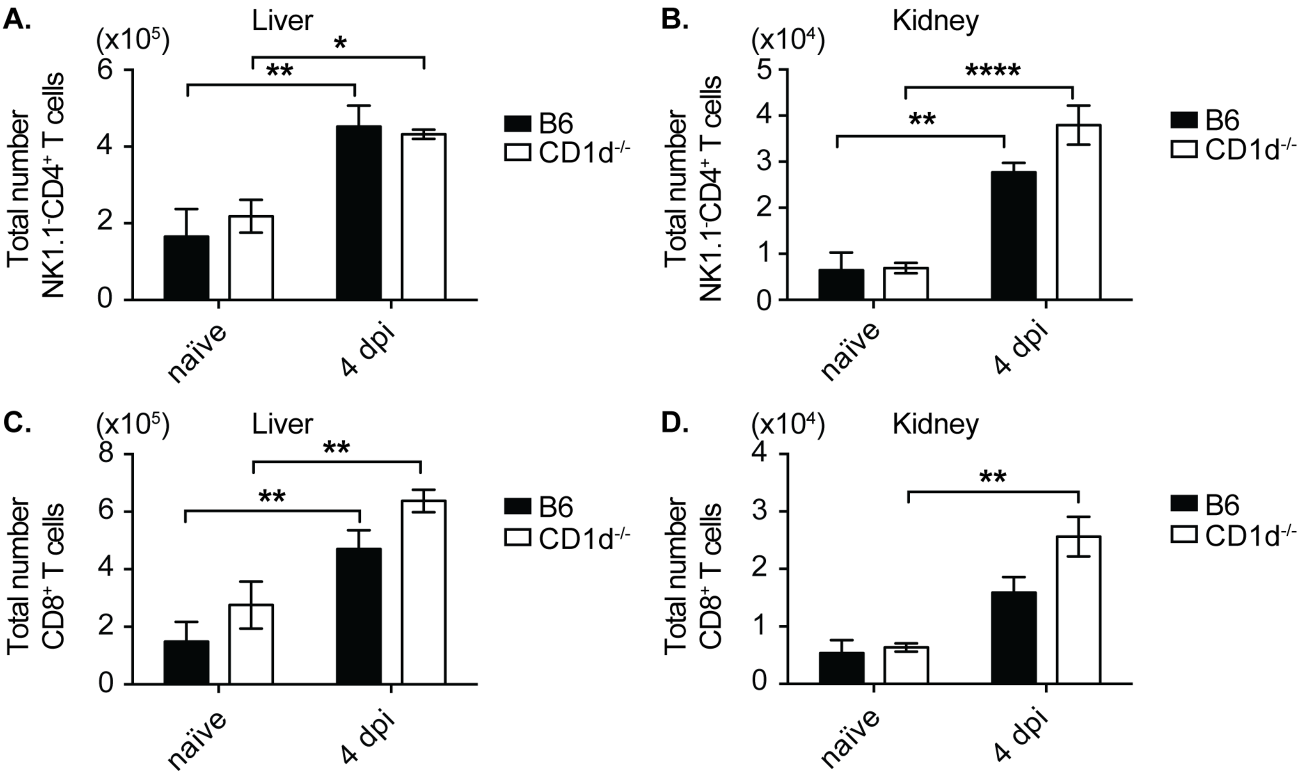

S2 Fig. Absence of NKT cells does not alter conventional T cell expansion in infected organs. Total number of conventional $CD4^+$ T cells and $CD8^+$ T cells from the liver (A, C) and kidney (B, D) at 4 dpi (N=3 naïve, N=7 4 dpi mice). Statistical analysis: 2-way ANOVA.

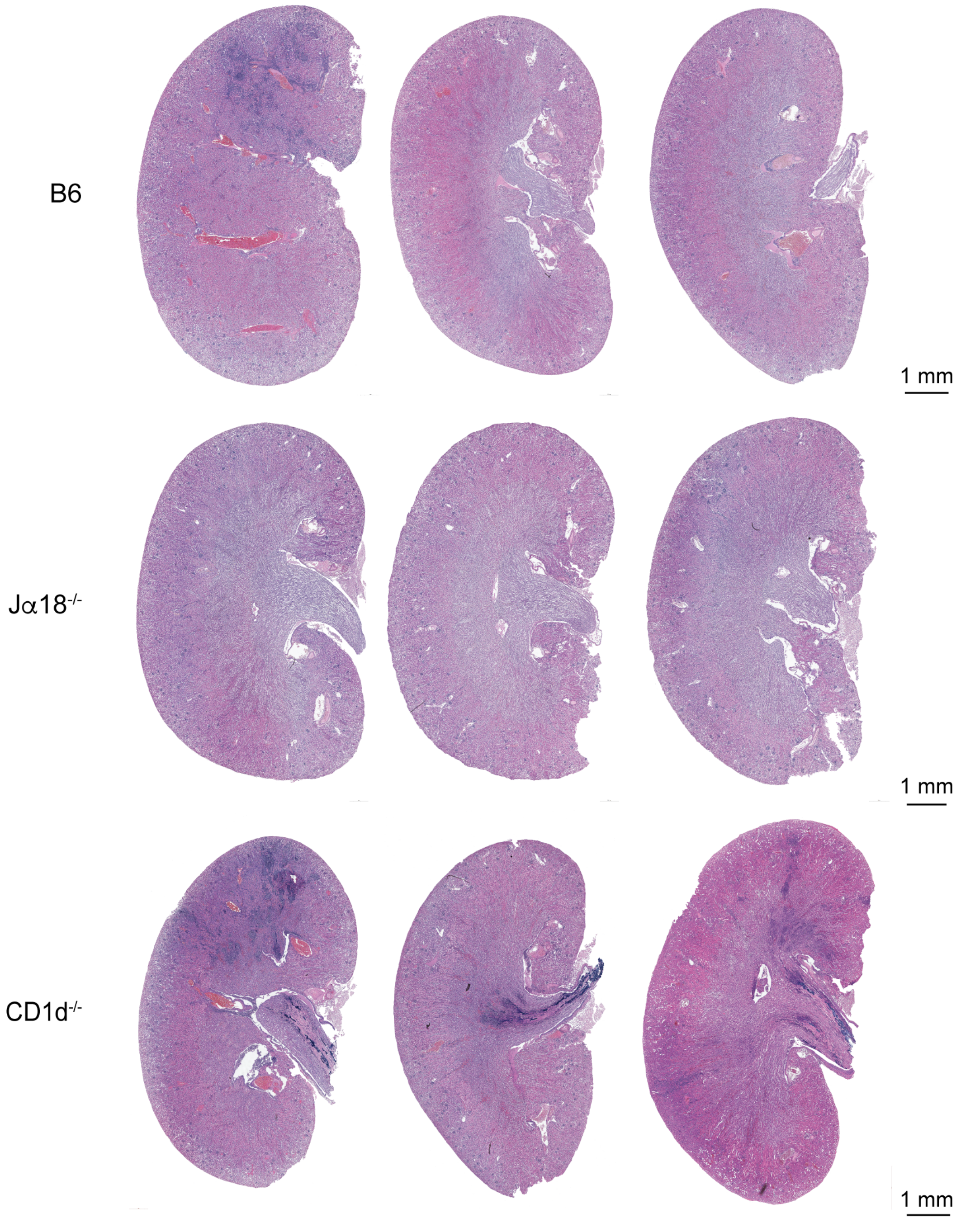

S3 Fig. H&E sections of quantified kidney inflammatory foci areas.

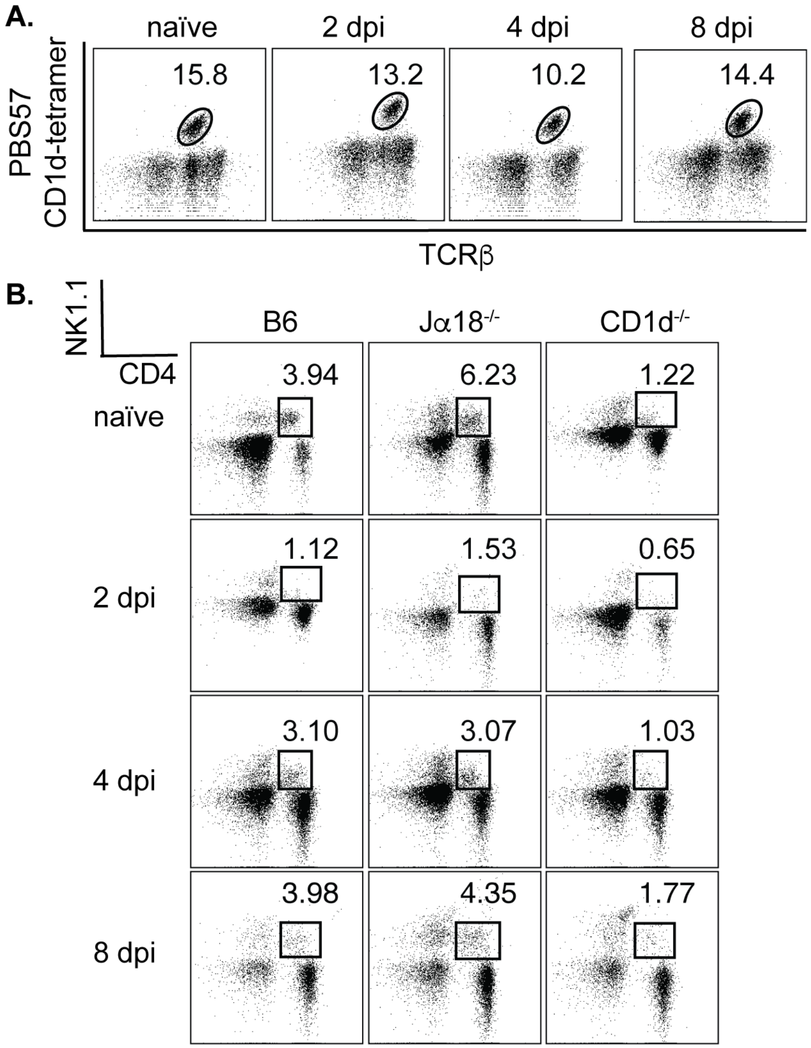

S4 Fig. Time course of NKT cell kinetics during SA infection. **(A)** Type I NKT cells gated in B6 liver. **(B)** Type II NKT cells gated in B6, $Ja18^{-/-}$, and $CD1d^{-/-}$ liver.

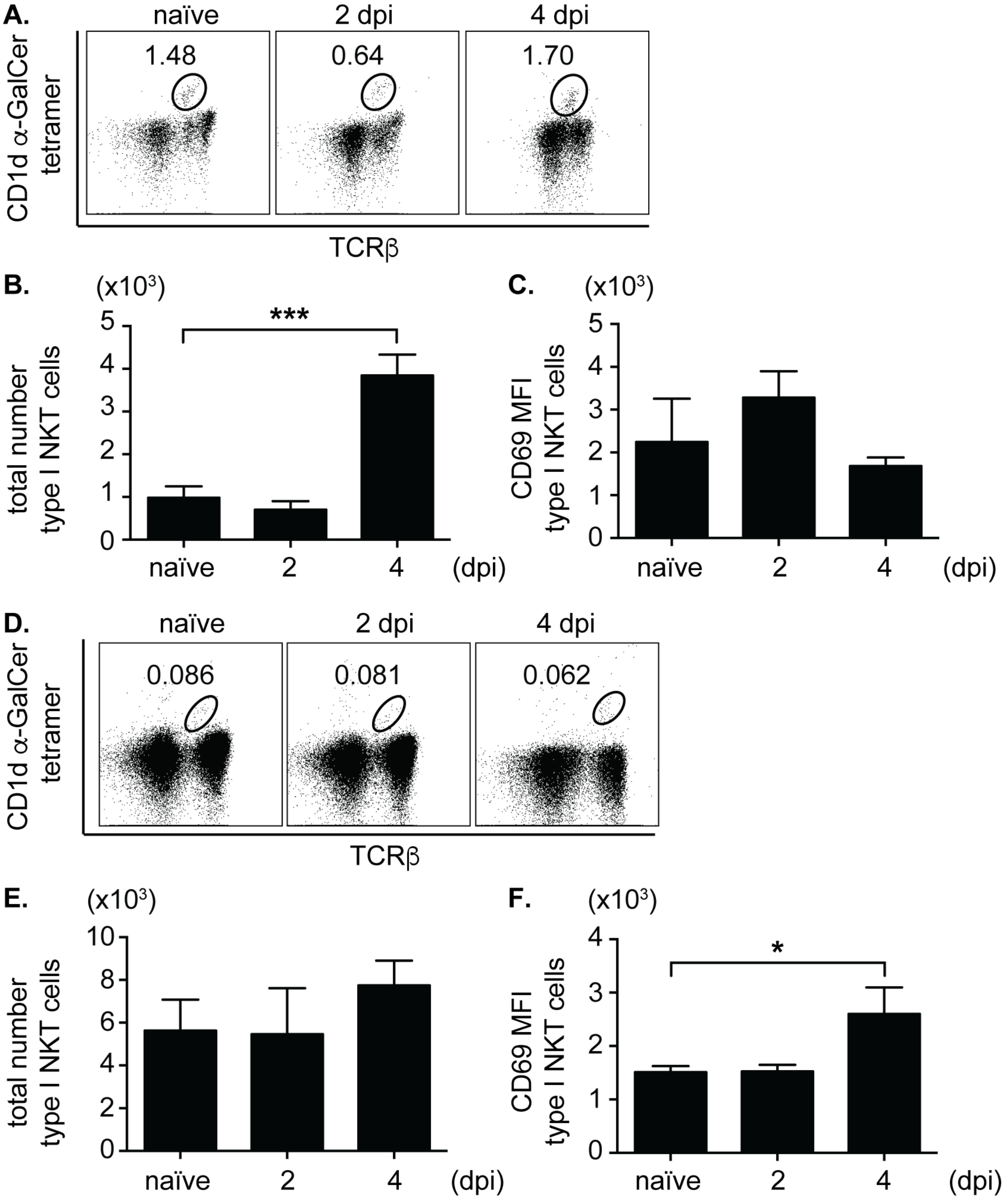

S5 Fig. Type I NKT cells are expanded in kidney, not lymph node, after SA infection.
(A) Representative FACS plots of type I NKT cells in the kidney of B6 mice at various times post infection. **(B, C)** Total cell number and CD69 MFI of type I NKT cells in the kidney of B6 mice (N=3 naïve, N=4-6 infected mice/timepoint). **(D)** Representative FACS plots of type I NKT cells in pooled peripheral and kidney draining lymph node (LN) of B6 mice at various times post infection. **(E, F)** Total cell number and CD69 MFI of type I NKT cells in LN of B6 mice (N=5 naïve, N=5-10 infected mice/timepoint). Statistical analysis: one-way ANOVA.

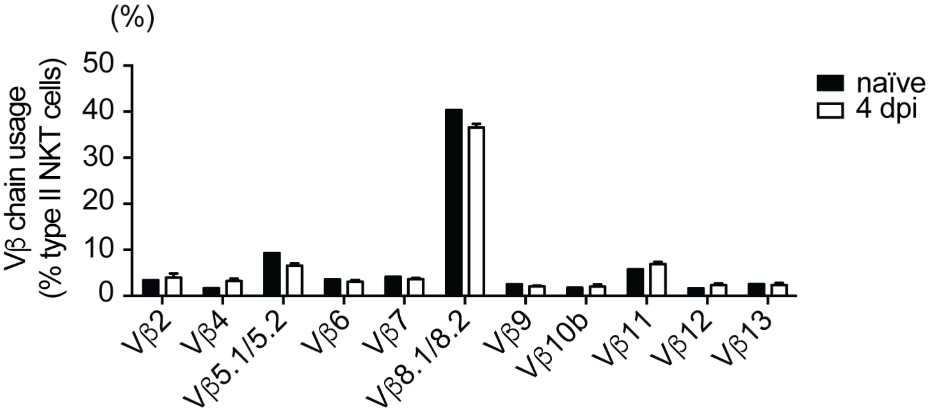

S6 Fig. Polyclonal type II NKT cells utilize diverse Vβ chains, which are unchanged after SA infection.

Vβ chain usage of type II NKT cells in the liver of Jα$18^{-/-}$ mice at 4 dpi (N=3 pooled naïve, N=7 infected mice). Statistical analysis: 2-way ANOVA.

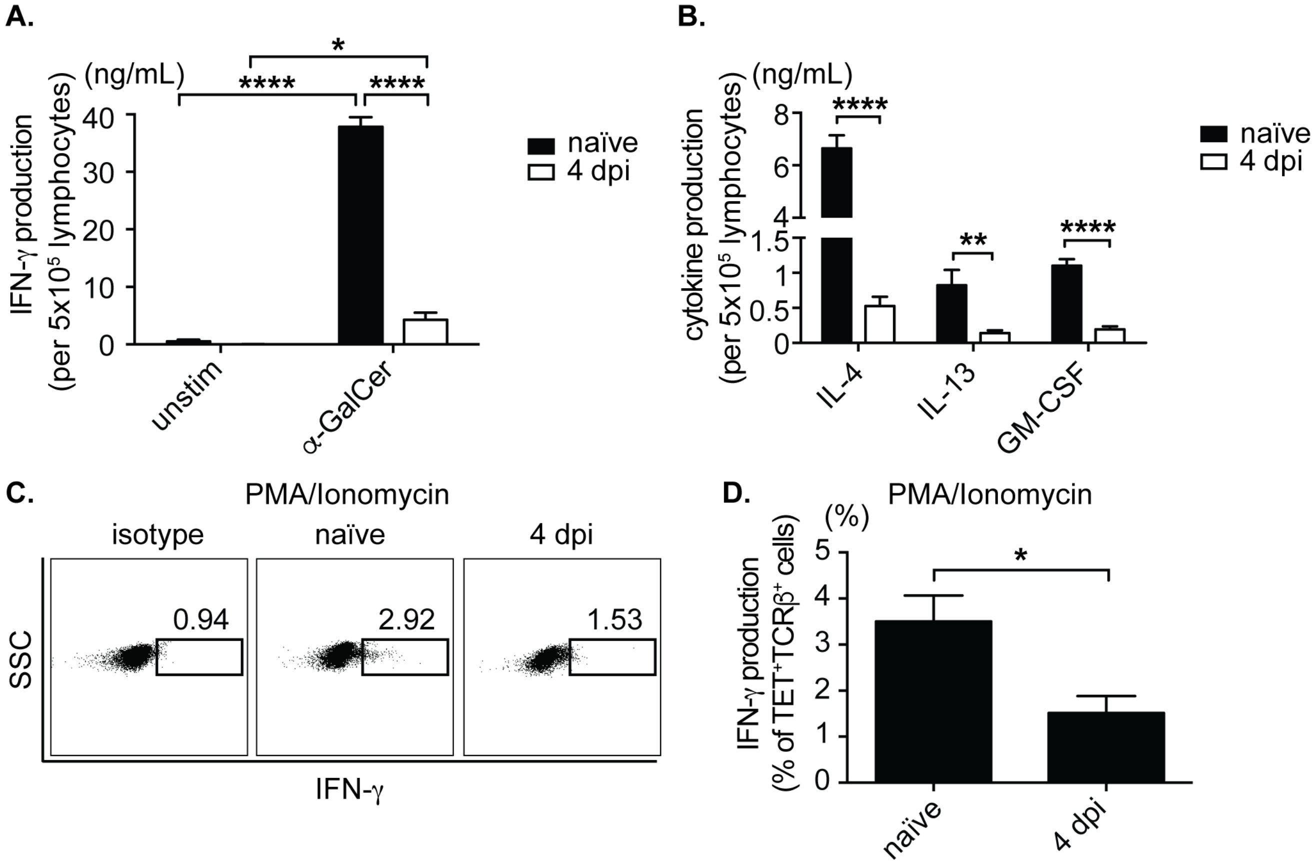

S7 Fig. Type I NKT cells are hyporesponsive to restimulation after SA infection.

(A) IFN-γ ELISA of B6 liver lymphocytes co-cultured with α-GalCer (N=2-5 mice/timepoint, representative 1 of 5). **(B)** CBA of B6 liver lymphocytes cultured with α-GalCer (N=2-5 mice/timepoint, representative 1 of 2). **(C, D)** ICS of type I NKT cells from naïve and 4 dpi mouse liver lymphocytes stimulated with PMA/Ionomycin (2 hrs + 4 hrs BFA) (C) and representative FACS plots (D) of IFN-γ producing cells represented as % of total type I NKT cells (N=4 naïve, N=5 4 dpi mice). Statistical analysis: (A, B) 2-way ANOVA, (D) student's t test.

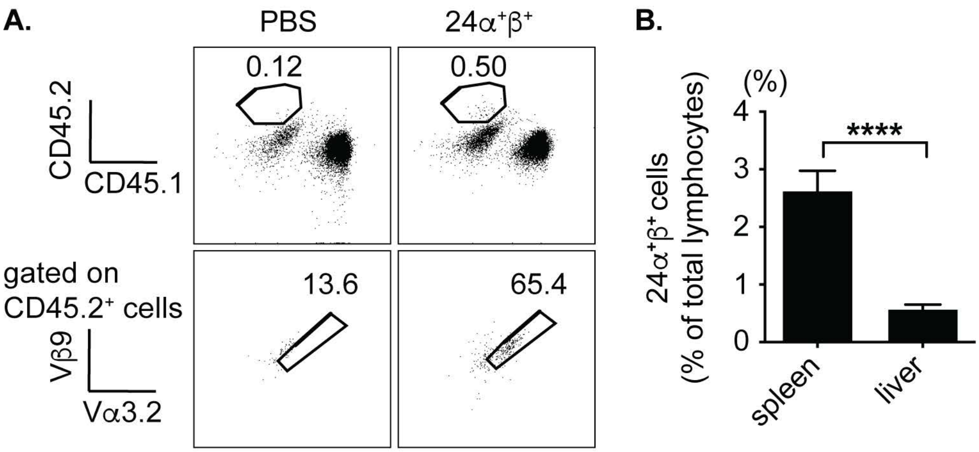

S8 Fig. 24α$^+$β$^+$ NKT cells were enriched in spleen relative to liver after adoptive transfer.

(A) Representative FACS plots of adoptively transfered 24α$^+$β$^+$ NKT cells from the liver of recipient CD45.1 mice at 2 dpi, PBS= control group. **(B)** 24α$^+$β$^+$ NKT cells in organs of recipient mice, represented as % of total lymphocytes from each organ (N=10 mice). Statistical analysis: (B) student's t-test.
